# Exhaled VOC detection in lung cancer screening: a comprehensive meta-analysis

**DOI:** 10.1186/s12885-024-12537-7

**Published:** 2024-06-27

**Authors:** Xianzhe Fan, Ran Zhong, Hengrui Liang, Qiu Zhong, Hongtai Huang, Juan He, Yang Chen, Zixun Wang, Songlin Xie, Yu Jiang, Yuechun Lin, Sitong Chen, Wenhua Liang, Jianxing He

**Affiliations:** 1 Department of Thoracic Surgery and Oncology, The First Affiliated Hospital of Guangzhou Medical University, Guangzhou, 510120 Guangdong China; 2 ChromX Health Co., Ltd, Guangzhou, Guangdong China; 3 Department of Thoracic Surgery and Oncology, State Key Laboratory of Respiratory Disease, National Center for Respiratory Medicine, The First Affiliated Hospital of Guangzhou Medical University, Guangzhou, Guangdong 510120 China

**Keywords:** Volatile organic compounds, Lung cancer, Exhaled, Diagnostic accuracy

## Abstract

**Background:**

Lung cancer (LC), characterized by high incidence and mortality rates, presents a significant challenge in oncology. Despite advancements in treatments, early detection remains crucial for improving patient outcomes. The accuracy of screening for LC by detecting volatile organic compounds (VOCs) in exhaled breath remains to be determined.

**Methods:**

Our systematic review, following PRISMA guidelines and analyzing data from 25 studies up to October 1, 2023, evaluates the effectiveness of different techniques in detecting VOCs. We registered the review protocol with PROSPERO and performed a systematic search in PubMed, EMBASE and Web of Science. Reviewers screened the studies’ titles/abstracts and full texts, and used QUADAS-2 tool for quality assessment. Then performed meta-analysis by adopting a bivariate model for sensitivity and specificity.

**Results:**

This study explores the potential of VOCs in exhaled breath as biomarkers for LC screening, offering a non-invasive alternative to traditional methods. In all studies, exhaled VOCs discriminated LC from controls. The meta-analysis indicates an integrated sensitivity and specificity of 85% and 86%, respectively, with an AUC of 0.93 for VOC detection. We also conducted a systematic analysis of the source of the substance with the highest frequency of occurrence in the tested compounds. Despite the promising results, variability in study quality and methodological challenges highlight the need for further research.

**Conclusion:**

This review emphasizes the potential of VOC analysis as a cost-effective, non-invasive screening tool for early LC detection, which could significantly improve patient management and survival rates.

**Supplementary Information:**

The online version contains supplementary material available at 10.1186/s12885-024-12537-7.

## Introduction

Currently, LC ranks among the cancers with the highest incidence rates. Despite a variety of treatments available that can prolong life and enhance quality of life, LC continues to be a predominant cause of cancer-related mortality [1]. Research extensively suggests that early screening and detection are the most effective strategies to reduce mortality and improve survival rates in LC patients [2]. Consequently, enhancing the efficiency of early LC diagnosis has emerged as a vital area of research. Improving the early diagnosis rate of LC necessitates advancements in screening techniques. At present, radiological examinations, particularly low-dose computed tomography (LDCT), are deemed the most effective methods for LC screening. However, the high false-positive rate, unavoidable radiation exposure, and significant costs associated with LDCT limit its broad application [3].

In recent years, molecular biology approaches have received increased focus in cancer screening. These include the detection of tumor markers in bodily fluids, such as carcinoembryonic antigen (CEA) and circulating tumor DNA (ctDNA). Nonetheless, these methodologies are limited in sensitivity, and the search for novel, specific biomarkers is ongoing and challenging [4]. To address these limitations, a novel method involving the detection of VOCs in patients’ breath has been proposed.

Human physiological processes yield a multifaceted array of metabolic byproducts, which may originate directly within the lungs or be conveyed to the lungs via the bloodstream, subsequently being expelled through gaseous exchange. The contrast in physiological activities between tumoral and normal tissues results in distinct metabolic byproducts. By identifying these differentially produced compounds, it is viable to screen for prospective lung cancer patients [5]. Obtained via exhalation, these VOCs can thereafter be analyzed through two fundamental techniques: Chemical compound analysis employing gas chromatography and mass spectrometry (GC–MS), which allows for the examination of individual compounds, or the application of an electronic nose (eNose) that utilizes pattern recognition of chemical compounds through multivariate analysis. This burgeoning technology presents a promising adjunct, proffering a simple, swift, non-invasive, point-of-care diagnostic tool potentially amenable to widespread screening efforts, ultimately aimed at refining lung cancer management strategies. Consequently, a systematic review of pre-existing studies was undertaken to ascertain the viability of employing volatile organic compounds in lung cancer screening.

## Materials and methods

### Eligibility criteria

**Fig. 1:**
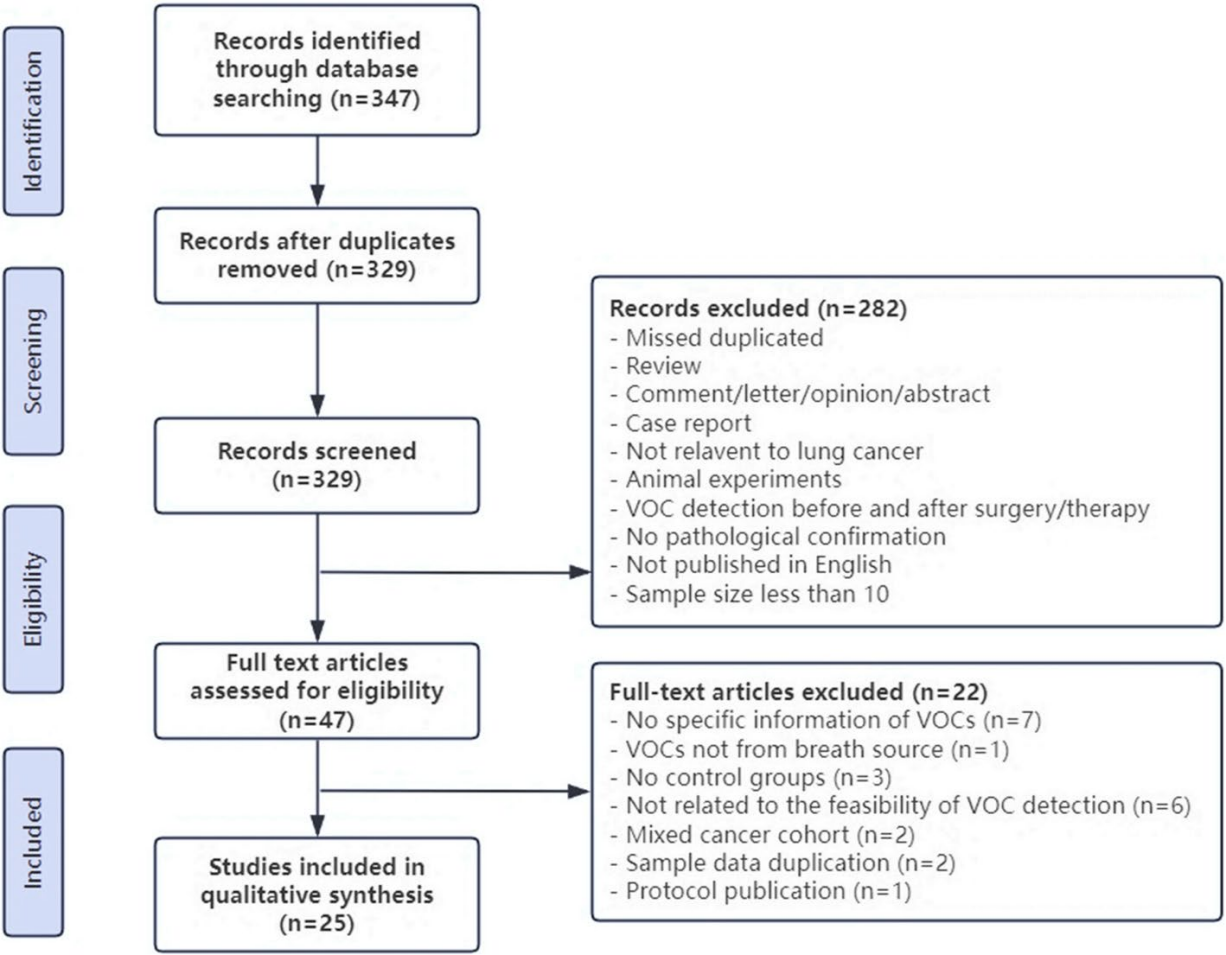
Flowchart of selecting eligible studies

The inclusion criteria for controlled trials were as follows: patients diagnosed with LC via pathological or cytological confirmation; detection of volatile organic compounds (VOCs) in the exhalation of the subjects; and clinical studies. Exclusion criteria included studies with small sample sizes; lack of a healthy control group; focus on VOC detection technology; studies on VOC changes before and after LC treatment; studies not reporting the detected VOC results; and articles not written in English or unpublished. The detailed patient data for our analysis are shown in Appendix Table 2 (Fig. 1).

### Search and study selection

This meta-analysis was conducted in accordance with PRISMA guidelines. Two experienced reviewers (X.F. and Z.R.) searched the PubMed, EMBASE, and Web of Science databases for literature published up to October 1, 2023. The keywords used were “lung cancer,” “volatile organic compounds,” and “exhalation screening” and the appropriate Medical Subject Heading (MeSH) terms. A specific search strategy was employed for each database, and the protocol was registered with PROSPERO (Registration No. CRD 42023470519).

### Data extraction

In addition to basic information such as authors and publication dates, we extracted as much relevant information as possible about the experimental and control groups from the articles. All data related to the target outcomes were recorded in a Microsoft Excel database. This included author names, publication years, data on the subjects of the experimental and control groups, VOC detection methods, and the diagnostic performance of exhaled VOC detection (sensitivity and specificity). Since almost all articles did not provide the number of true positives (TP), false positives (FP), true negatives (TN), and false negatives (FN), these metrics were calculated using the sensitivity, specificity, and the number of subjects in the experimental and control groups provided in the literature.

### Assessment of study quality

The quality of the included studies was assessed using the QUADAS-2 tool, which evaluates four key domains: patient selection, index tests, reference standards, and the flow and timing of the studies. Each component was assessed for risk of bias, with the first three domains also evaluated for their clinical applicability. The outcomes of this assessment were categorized into three risk levels: low, high, and unclear (Appendix Table 4, Appendix Fig. 1). The patient selection domain, predominantly based on case–control study designs, exhibited higher risks of bias and applicability. Similarly, most studies had high bias risks in the index test domain due to the reliance on known pathological results as the reference standard, though the applicability concerns here were minimal. Nearly all studies employed pathological examination as their reference standard, leading to low bias risks and high applicability in this domain. The majority of studies presented a low bias risk regarding study flow and timing, except for three studies that did not clearly specify whether all patients underwent the same testing standards, resulting in unclear bias risks, and three others that either did not use uniform testing standards or failed to include all cases in the analysis, thus posing a higher risk.

## Results

### Study selection and characteristics

Subsequent to the elimination of duplicates, 329 studies were earmarked for screening. Amongst these, 304 were dismissed for failing to conform to the prescribed inclusion criteria, resulting in the selection of 25 studies for incorporation into the systematic review. This review encapsulates 25 investigations centered on the utilization of VOCs detected in exhalations for screening LC, encompassing a cumulative cohort of 2045 individuals diagnosed with LC and 2201 subjects in the control group, which included both healthy individuals and those diagnosed with benign respiratory conditions. The contributing studies spanned 10 countries, with a preponderance originating from the United States and China. Given the constraints of small sample sizes, the majority of studies resorted to cross-validation methods for verification. The collection of exhalation samples predominantly employed Tedlar bags, although a subset of studies utilized sorbent traps among alternative methodologies. Eighteen investigations adopted gas chromatography-mass spectrometry (GC–MS) as the analytical technology, notably including the study by Wang et al., which implemented a synergy of SPME and TD techniques in conjunction with GC–MS. An additional two studies leveraged GC-FID technology, whereas five other investigations utilized a diverse array of technologies, including IMS, SIFT-MS, HPPI-TOFMS, electronic nose (eNose), and CRDS. The compilation of VOCs discerned across these studies illuminated 37 compounds recurrent in three or more investigations, posited as potential biomarkers for LC screening. These VOCs were categorized according to prevalence, spanning alkanes, alkenes, ketones, benzenes and their derivatives, aldehydes, and alcohols, with hexanal emerging as the most prevalently detected compound. The sensitivity range deployed for LC screening via VOCs oscillated between 60.6% and 100%, while specificity ranged from 61.2% to 100%.

### Meta-analysis

**Fig. 2:**
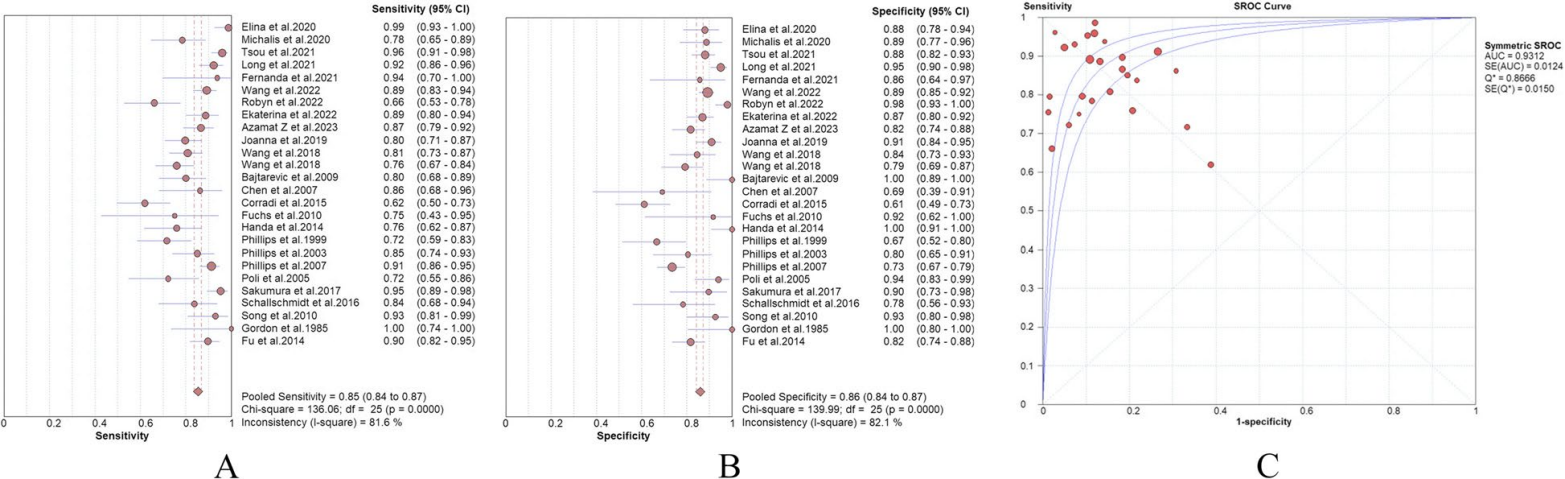
Clinical sensitivity (**A**), specificity (**B**) and SROC curve (**C**) of VOCs detection methods in the included studies

According to the retrieved data, we estimated the accuracy of VOCs as a screening tool for LC. The meta-analysis of exhaled VOCs revealed the pooled sensitivity and specificity of all the included studies were 85% (95% CI 84–87%) and 86% (95% CI 84–87%) (Fig. 2A, B), respectively The SROC curve revealed an AUC of 0.93 (Fig. 2C), indicating outstanding diagnostic performance. And the PLR, NLR, and DOR were 6.10 (95% CI 4.61–8.05), 0.18 (95% CI 0.13–0.23) and 42.26 (95% CI 25.68–69.55), respectively (Appendix Fig. 2A, B, C). Due to the scarcity of studies analyzing the correlation between LC staging, subtypes, and exhaled VOCs, such data were limited and challenging for statistical analysis. The software used for the meta-analysis included Excel, Origin 2021, and Meta-disc.

## Discussion

The findings of this review suggest that VOCs present in exhaled breath may represent a novel and promising method for LC screening. This analysis encompassed 25 studies involving a total of 2045 LC patients and 2200 control group participants. Table 1 summarizes the essential information. The sensitivity and specificity for the detection of LC using exhaled VOCs were reported to be 85% (95% CI: 84–87%) and 86% (95% CI: 84–87%), respectively, with an AUC of 0.93. These findings demonstrate the high diagnostic accuracy of breath analysis for LC. However, given the significant risk of bias in the assessment of research quality, these results warrant cautious interpretation.

**Table 1:**
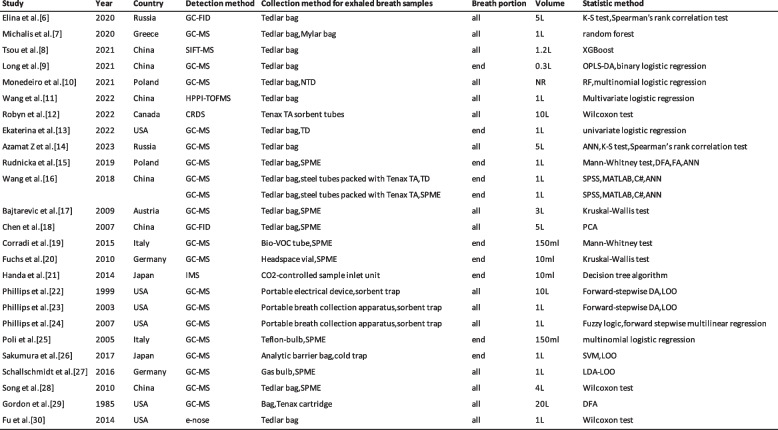
Characteristics of included studies [6–30]

*GC* gas chromatography, *MS* mass spectrometry, *FID* flame ionization detector, *SIFT* selected ion flow tube, *NTD* Needle Trap Device, *HPPI-TOFMS* high-pressure photon ionization time-of-flight mass spectrometry, *CRDS* cavity ring-down spectroscopy, *TD* thermal desorption, *IMS* Ion mobility spectrometry, *XGBoost* eXtreme Gradient Boosting, *OPLS-DA* orthogonal projection to latent structure discriminant analysis, *RF* random forest, *ANN* artificial neural network, *DFA* discriminant function analysis, *FA* factor analysis, *PCA* Principal component analysis, *LOO* Leave-one-out cross-validation, *SVM* Support vector machine, *LDA* Linear discriminant analysis

Current mainstays for LC screening include LDCT and serum biomarkers. LDCT, extensively utilized, has been validated in previous studies to decrease LC mortality and enhance quality of life [31]. Nevertheless, despite its high sensitivity, LDCT’s specificity is comparatively low, and there is a potential risk of radiation damage, particularly affecting the elderly. Moreover, utilizing LDCT for screening may elevate the incidence of radiation-induced LC [3]. Serum biomarkers for LC, such as CEA, CYFRA 21–1, and ctDNA, are commonly employed. However, their sensitivity and specificity leave much to be desired. In contrast, the detection of VOCs in exhaled breath offers simplicity, non-invasiveness, and lacks radioactive exposure, thereby enhancing patient compliance and achieving optimal detection accuracy. Consequently, this method holds considerable potential for further development and widespread application.

### Chemicals classes

**Fig. 3:**
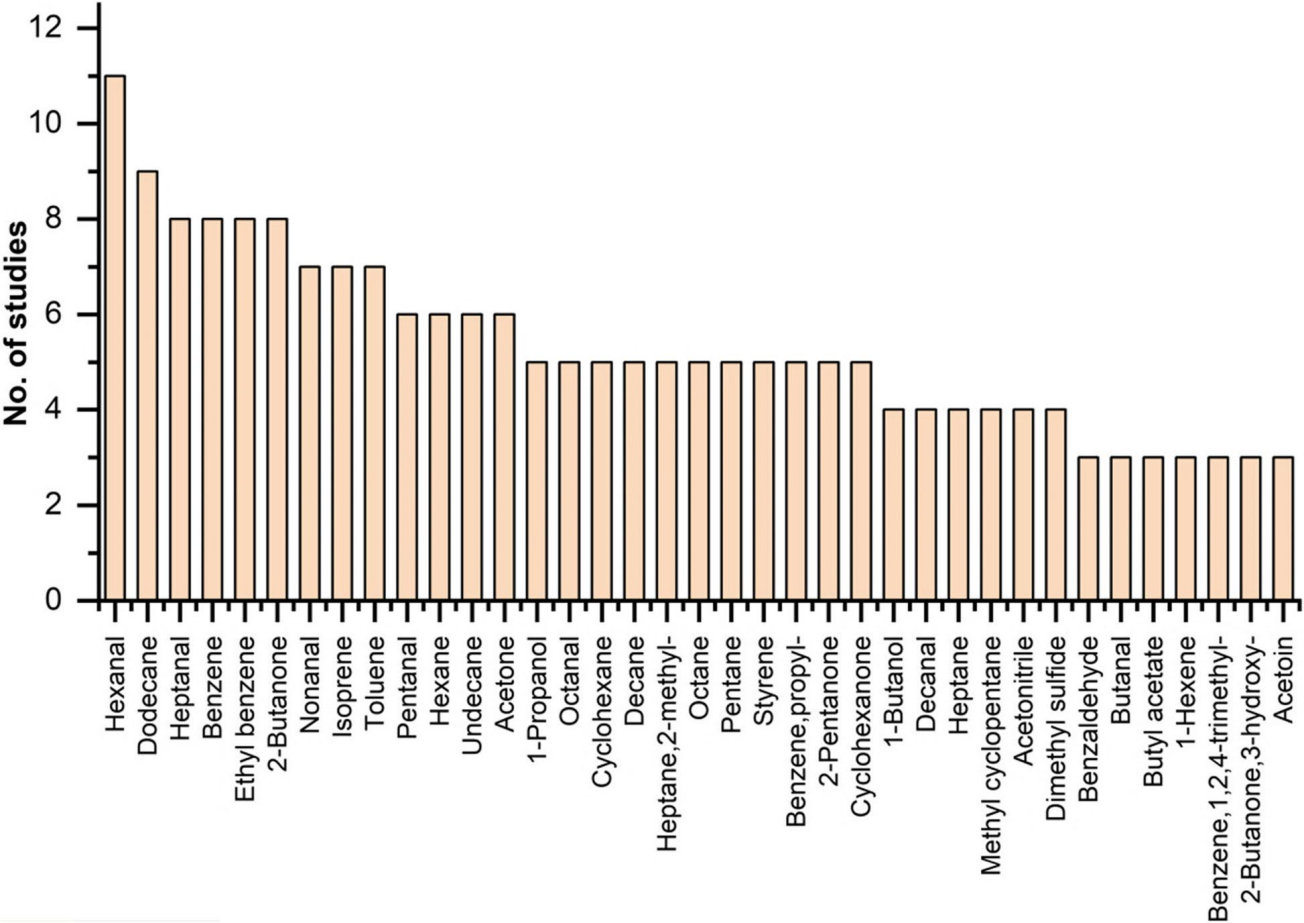
The most frequent detected VOCs for LC in studies

A total of 190 VOCs were detected in the breath of LC patients in the included studies (Appendix Table 6). However, only 37 VOCs were detected in 3 or more articles among the 7 detection methods (Fig. 3), highlighting a low reproducibility of VOC detection across different studies. Employing consistent or standardized detection methods might help mitigate this issue. The compounds identified primarily comprise alkanes, alkenes, ketones, benzene, aldehydes, and alcohols, with hexanal being the most prevalent, detected in nearly half of the studies. Developing a specific VOC spectrum based on these frequently mentioned compounds could significantly enhance the diagnostic efficacy for LC.

The most commonly detected compounds in the included studies were alkanes, followed by alkenes, both of which belong to hydrocarbons. Under normal physiological conditions, human cells produce a certain amount of reactive oxygen species (ROS), which can cause peroxidation of lipids, especially polyunsaturated fatty acids (PUFA), when they are abnormal. This is one of the main sources of hydrocarbons [32]. Pentane, one of the most frequently detected alkanes, was once considered a marker of lipid peroxidation [33], but pentane can be further metabolized into 2-pentanol in the liver, so factors that affect the liver may change the concentration of pentane [34]. Isoprene appeared with the highest frequency among alkenes, and research has shown that it is mainly produced in the liver through the mevalonate pathway (MVA) from cholesterol biosynthesis [35]. However, other studies have confirmed that there is no direct correlation between the concentration of isoprene in breath and blood cholesterol levels [36, 37], so there may be other biological pathways that affect the concentration of isoprene in breath.

The β-oxidation process of long-chain fatty acids in mitochondria is the basis for ketones and ketone derivatives in the human body. The active metabolism of fatty acids in cancer is also the main reason for the increase in ketone content in patients’ breath [32, 38]. Acetone is the simplest ketone with a high detection rate. However, its concentration is easily affected by metabolic diseases such as diabetes and physiological activities, so it’s potential as a biomarker still needs to be considered [39]. Benzene and its derivatives are generally believed come from the external environment, such as air pollution, cigarettes, chemical materials, etc. [40]. In fact, the aromatase which synthesizes hormones, is overexpressed in human breast cancer tissue, which may lead to changes in the concentration of benzene in the VOC spectrum [41], whether this phenomenon exists in LC needs further explore.

Hexanal is the most frequently detected substance among all tested substances, with 11 studies mentioning it, indicating its importance as a potential LC biomarker. The sources of aldehydes and alcohols are very diverse, including diet, smoking, alcohol intake [42]. They are closely related to the metabolism of multiple types of substances. For example, the metabolism of hydrocarbons can produce alcohols, the oxidation reaction of alcohols in the liver can produce aldehydes, and CYP450 can also participate in the oxidation of alcohols to produce aldehydes [43].

The sources of esters, ethers and furan, in addition to dietary intake, mostly rely on enzymatic reactions [44]. Nitrogen and sulfur compounds mainly originate from the decomposition and synthesis of specific amino acids [45, 46]. However, more researches are needed in the future to prove whether they have potential to become biomarkers due to the low existence in breath.

In fact, only a portion of the most frequent compounds detected in the included studies have clear origins (Table 2), The majority of VOC sources are still unclear or only have some hypothetical origins. Moreover, even if some substances have a clear origin, their differences in LC patients may not necessarily indicate their ability to serve as biomarkers. For example, lung infections, non-specific inflammation, and other tumors also exhibit oxidative stress, and most inflammatory conditions are related to the production of ROS. Therefore, some ROS products may not be specific to cancer [42]. Many studies have included other lung diseases, such as benign pulmonary nodules, COPD, and LC for comparison to screen specific VOC. Some common non-tumor and non-inflammatory metabolic diseases will also lead to changes in VOC [47]. Smoking is one of the most important risk factors for LC, it is still uncertain whether it has an impact on respiratory test results. Phillips et al.’s study [23] suggests that smoking has no significant effect on VOC spectra, while Long’s [9] and Corradi’s study [19] provide the opposite conclusion. This also confirms that the accuracy of using a single VOC diagnosis is low. While searching for specific biomarkers, it is also necessary to establish a diagnostic model that combines multiple VOCs. More research should be invested to explore the origin and related influencing factors of these substances, so that the clinically relevant information obtained from respiratory analysis is meaningful.

**Table 2:**
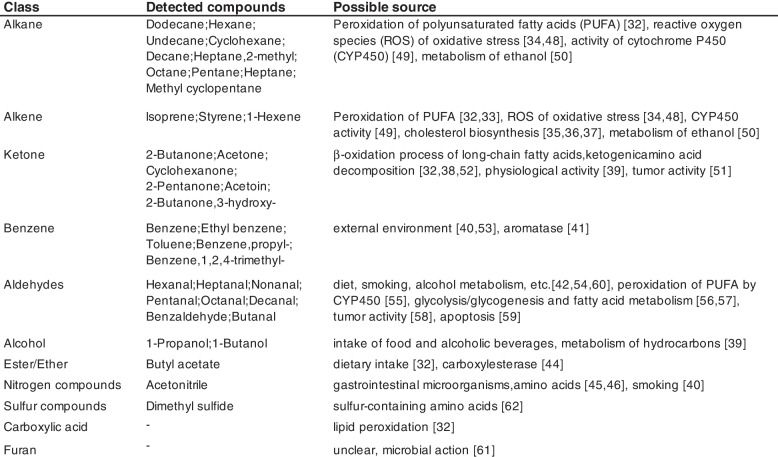
The most frequent detected VOCs and their possible sources [32–46, 48–62]

### Stage and histological type

The baseline data of the subjects are shown in Table 3. Many of the included studies recorded the LC stage and VOCs spectrum, but few further explored the relationship between them, nor do they set subgroups by histological type. In an instructive comparison conducted by Fuchs et al. [20], an analysis on VOC spectra between SCLC and NSCLC exhibited an elevated concentration of hexanal in SCLC (*p* = 0.006), presumably attributed to its higher malignancy and augmented tumor cellular activity. Handa et al. [21] found that EGFR mutations could lead to changes in VOC spectra, especially the n-Dodecane peak (*p* < 0.01). The study also confirmed that 2-Butanol, 2-Methylfuran, and n-Nonanal could also be used to distinguish adenocarcinoma from squamous cell carcinoma (*p* = 0.011). Song et al. [28] observed that adenocarcinoma had higher levels of 1-butanol and 3-hydroxy-2-butanone than squamous cell carcinoma (*p* < 0.05), while Fu et al. [30] reported an ascendance in the 4-hydroxyhexenal peaks within the VOC spectrum of squamous cell carcinoma (*p* = 0.03). Different types of tumor cells produce diversity in VOCs profiles due to their differences in biological behavior. Chen’s cytological experiment [18] also proved that different types of LC cells have their own unique VOC spectra. Associative studies have highlighted LC staging correlations with VOCs spectral changes, where Chen [18] found that the VOC species of patients with stage I, II LC were the same as those of patients with stage III, IV LC, but the concentrations were different, suggesting that VOC detection is more suitable for stage I, II. LC. Fu et al. [30] found that the concentration of 2-butanone was significantly lower in patients with stage I LC than in patients with stage II to IV LC. Conversely, Azamat et al. [14] postulated an increased concentration of 2-butanone in all advanced LC stages. Azamat also found that peaks such as 2,3-Butandione and 1-methylthiopropene were significantly correlated with TNM staging of LC. Corradi et al.’s study [19] found that the concentration of ethyl benzene increased in the breath of patients with advanced NSCLC (*p* = 0.019). On the contrary, several studies by Phillips [22–24] and Song et al. [28] suggested that the stage of LC had little impact on breath VOC detection. Although these studies analyzed the correlation between LC staging and VOC changes, the number of studies remains small, and the number of samples included in the studies is also small. Some research conclusions are conflicting, so further exploration is needed in this area in the future. Handa proposed establishing a unique VOC profile based on ethnicity, age, and other factors [21], which would also be a feasible research direction.

**Table 3:**
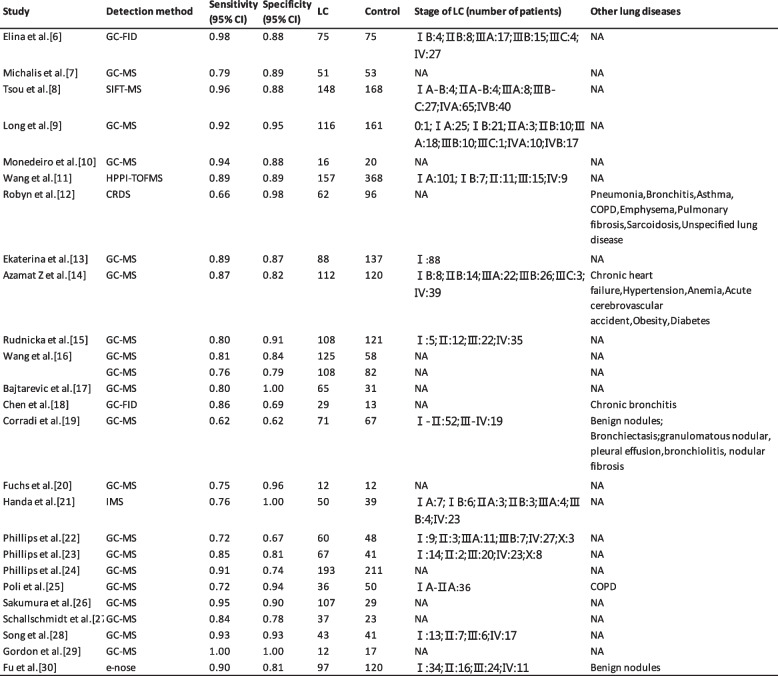
Baseline information of people in the studies [6–30]

### Collection methods

The collection of breath samples is an indispensable step in the detection of VOCs. Tedlar bag is the most commonly used collection method. Considering the low concentration of target VOCs in the sample and susceptibility to environmental contaminants, sample preparation often involves the combined use of solid phase microextraction (SPME), sorption tubes, or thermal desorption (TD) methods. Wang et al. [16] compared two sample preparation schemes, TD and SPME, extrapolating a diagnostic model sensitivity, specificity, and accuracy of 80.8%, 84%, and 82.7% respectively for GC–MS in conjunction with TD; and 75.6%, 78.9%, and 76.7% with GC–MS integrated with SPME. In addition, some literature has also attempted to use new collection methods, such as Monedeiro et al.’s [10] use of Needle trap device (NTD). This method effectively reduces the limitations of sample collection and quantification, greatly improving sample utilization and research analysis reproducibility. Several of the studies included end-tidal breath samples for analysis. Generally, the first two-thirds of human exhaled breath consist of airway gases, while the final one-third comprises alveolar gases. The VOC spectrum found in alveolar gases is relatively less impacted by external environments compared to airway gases, resulting in VOC concentrations that are 2–3 times higher than those in the total breath [63], which may render them more suitable for analysis. Moreover, the volume of samples collected in the study ranged from 10 ml to 10L, indicating a need for further research to ascertain if sample volume impacts detection outcomes. In summary, the collection method of samples does influence the accuracy of VOC detection, so reforming old technologies and developing new ones should be emphasized.

### Detection methods

In fact, GC–MS is currently recognized as the gold standard for biomarker identification in human breath [64]. It can accurately analyze the specific compound in breath, but the method’s prohibitive cost, sophisticated equipment requirements, and steep learning curve hinder its widespread application in LC screening. Some of the literature included in this study used other detection methods, such as GC-FID and SIFT-MS. GC-FID, leveraging a flame ionization detector (FID), and SIFT-MS, utilizing a selective ion flow tube (SIFT), represent advancements over the traditional GC–MS approach, convincingly enhancing LC screening sensitivity. E-nose and high-pressure photon ionization time-of-flight mass spectrometry (HPPI-TOFMS) have demonstrated comparative accuracy in LC screening to that of GC–MS. While neither method quantitatively measures VOC concentrations akin to GC–MS, they provide alternative indicators, such as compound mass spectra peaks. Furthermore, these two methods can be used as portable devices for clinical real-time detection without sample preprocessing, reduced the possibility of contamination and enhance their portability. The other two detection methods Ion mobility spectrometry (IMS) and cavity ring-down spectroscopy (CRDS) exhibited lower accuracy levels. IMS obtains a single spectrum with a certain time limit and is generally not used for unknown substance detection, making it difficult to discover new LC biomarkers in breath; CRDS is a laser absorption spectroscopy technology based on the principle of laser pulse decay in samples, with high specificity but low sensitivity. Although there are many different detection schemes available, the paucity of extensive research beyond GC–MS underscores a compelling need for further investigation into their practical viability.

### Statistical methods

Research has used various statistical methods for screening LC using VOCs. Traditional methods include t-tests, Wilcoxon tests, Mann–Whitney-U tests, principal component analysis, discriminant analysis (DA), etc. These methods are mostly used to analyze the linear relationship between VOCs and LC. However, given the complex interrelations among different VOCs, traditional algorithms do not fully utilize VOC data, potentially ignoring some nonlinear relationships [65]. In recent years, machine learning techniques have gradually become popular, such as random forest algorithms (RF) and artificial neural networks (ANN), which have gradually been introduced into research, promising more profound insights into VOC data interconnections. However, the establishment of these diagnostic models requires a large amount of data support, and the number of participants involved in the studies included is relatively small. Many studies also divide training and testing groups from the same sample dataset, so the fitted diagnostic models are mostly not ideal. Robyn et al. pointed out that overfitting models can overestimate or conservatively estimate the actual test performance [12]. Therefore, increasing sample sizes or adopting novel statistical methodologies is advisable to improve the establishment of diagnostic models.

### Suggestions

The method of detecting LC through the content of VOC in breath has been proven to be feasible. However, compared with other established and widely-used detection methods, significant gaps remain. The detection methods used in the included studies are different, as are the sampling collection, data statistics, and selection of target VOCs. These problems greatly limit the wide application of VOC detection. Therefore, establish standards to homogenize breath testing protocols is imperative. Many studies only differentiate VOC spectra between patients and healthy people and use this to evaluate the accuracy of breath testing,without established a standard for the so-called “standard VOC spectrum”. Since collecting samples from healthy people is feasible, establishing a corresponding “standard normal VOC spectrum” by age, race, etc. could lay a foundation for future research and clinical applications. In addition, there are two main directions for improving the accuracy of breath testing: developing new VOC detection technologies and improving the technologies already existed, or trying to find more specific LC biomarkers. Since the VOC contained in breath samples are easily affected by many aspects such as patient LC progression, lifestyle habits, and other diseases, detailed patient stratification and analysis of LC staging, typing, smoking habits, other lung conditions, and VOC spectra are necessary. The complexity of VOC interactions makes it difficult to apply a single VOC as a diagnostic standard, thus, VOC spectra should be used for comprehensive diagnosis. Evaluating compounds as potential characteristic biomarkers warrants consideration from diverse perspectives. Beyond sensitivity and specificity, factors such as compound stability, concentration in exhalation, and other relevant parameters should also be taken into account [66]. Future research may also incorporate cytological experimental studies to explore the biological characteristics of LC tumor cells and their unique VOC spectra, alongside clinical data to develop more effective diagnostic models. In addition, combining breath testing VOC with traditional LC screening methods such as CT can complement each other and may become a promising direction for future development.

### Limitation

Although we make a comprehensive evaluation of the exhaled VOCs detection, and the included studies have excellent applicability, our study still has certain limitations. 11 of the 25 included studies did not mention the stage of LC patients, and there were few subgroup analyses of patient staging in the recorded articles. Considering that LC staging may affect the changes in VOC spectra, our study cannot provide a conclusive answer. In addition, 9 articles did not mention the classification of LC patients, and although the remaining articles recorded the histological classification of patients, there was no unified classification method, and few articles studied the impact of different classifications on VOC spectra. Therefore, this study can only provide a general description of the feasibility of breath testing, but cannot provide specific clinical breath testing accuracy for LC. A total of 7 detection methods were included in the studies, but 19 studies used the same method GC–MS, which may affect our evaluation of the accuracy of other detection methods. In fact, besides GC–MS and eNose, using canine olfaction is also a cutting-edge but unconventional research direction [67]. However, considering individual differences in dogs and the inability of this method to quantitatively analyze specific VOCs, our study did not include articles in this research direction, and the induction of methods for using VOCs in exhaled breath to screen for lung cancer is not comprehensive enough.

## Conclusion

Our study included the latest research results on VOCs screening for LC in recent years, consistent with previous research conclusions, VOCs detection has excellent accuracy. This detection method exhibits rapidity, non-invasiveness, and significant patient adherence, rendering it highly promising for clinical application. Some compounds such as alkanes show a high correlation with LC, indicating use specific VOCs to construct models for diagnosing LC has high practicability. In addition, analyses were conducted on the differences in research results as well as the reasons for their occurrence, we also proposed some possible improvement plans. However, considering the existing researches still have some deficiencies, the factors that may affect the exhaled VOCs are still subject to various limitations that need to be further analyzed and verified. Therefore, in the future, it’s necessary to conduct research on a large number of population samples to further investigate their associations and explore more VOCs with the potential to become biomarkers for lung cancer. The research findings of this review may provide new supplements to the direction of improving exhaled breath detection.

### Supplementary Information

Supplementary Material 1.

## Data Availability

No datasets were generated or analysed during the current study.

## Abbreviations

LC: Lung cancer
VOC: Volatile organic compounds
GC: Gas chromatography
MS: Mass spectrometry
