# Supplementary material for: Exhaled VOC detection in lung cancer screening: a comprehensive meta-analysis
Source: BMC Cancer. 2024 Jun 27;24:775. doi: 10.1186/s12885-024-12537-7 (PMC11212189; doi:10.1186/s12885-024-12537-7)
Supplement: Supplementary file 1 — Supplementary Material 1. [file 12885_2024_12537_MOESM1_ESM.docx]

**Appendix Table 1.** Search strategy - Pubmed

| Search | Query | Results |
| --- | --- | --- |
| #4 | **#1 AND #2 AND #3** | 209 |
| #3 | **"Lung Neoplasms"[Title/Abstract] OR "Lung Cancer"[Title/Abstract] OR "Lung Tumor"[Title/Abstract] OR "Non-Small-Cell Lung"[Title/Abstract] OR "Carcinoma, Non Small Cell Lung"[Title/Abstract] OR "Carcinoma, Non Small Cell Lung"[Title/Abstract] OR "Non-Small-Cell Lung Carcinoma"[Title/Abstract] OR "Non Small Cell Lung Carcinoma"[Title/Abstract] OR "Non-Small Cell Lung Carcinoma"[Title/Abstract] OR "Non-Small Cell Lung Cancer"[Title/Abstract] OR "Carcinoma, Non-Small Cell Lung"[Title/Abstract] OR "Non-small Cell Lung Cancer"[Title/Abstract] OR "Small Cell Lung Cancer"[Title/Abstract]** | 215,648 |
| #2 | **"Breath"[Title/Abstract] OR "Exhale"[Title/Abstract] OR "**[**Respiration**](https://www.ncbi.nlm.nih.gov/mesh/68012119)**"[Title/Abstract]** | 142,134 |
| #1 | **"Volatile Organic Compounds"[Title/Abstract] OR "Compounds, Volatile Organic"[Title/Abstract] OR "Organic Compounds, Volatile"[Title/Abstract] OR "Volatile Organic Compound"[Title/Abstract] OR "VOC"[Title/Abstract] OR "VOCs"[Title/Abstract] OR "Compound, Volatile Organic"[Title/Abstract] OR "Organic Compound, Volatile"[Title/Abstract]** | 23,901 |

**Appendix Table 2.** Search strategy - Embase

| Search | Query | Results |
| --- | --- | --- |
| #4 | #1 AND #2 AND #3 | 228 |
| #3 | '**Lung Neoplasms**'/exp OR '**Lung Cancer**'/exp OR '**Lung Tumor**'/exp OR (‘**Lung Neoplasms**’ **OR** ‘**Lung Cancer**’ **OR** ‘**Lung Tumor**’ **OR** ‘**Non-Small-Cell Lung**’ **OR** ‘**Carcinoma, Non Small Cell Lung**’ **OR** ‘**Carcinoma, Non Small Cell Lung**’ **OR** ‘**Non-Small-Cell Lung Carcinoma**’ **OR** ‘**Non Small Cell Lung Carcinoma**’ **OR** ‘**Non-Small Cell Lung Carcinoma**’ **OR** ‘**Non-Small Cell Lung Cancer**’ **OR** ‘**Carcinoma, Non-Small Cell Lung**’ **OR** ‘**Non-small Cell Lung Cancer**’ **OR** ‘**Small Cell Lung Cancer**’):ti,ab,kw | 335,084 |
| #2 | 'breath'/exp OR (‘**Breath**’ **OR** ‘**Exhale**’ **OR** ‘[**Respiration**](https://www.ncbi.nlm.nih.gov/mesh/68012119)’):ti,ab,kw | 672,537 |
| #1 | 'volatile organic compound'/exp OR (‘VOC’ OR ‘VOCs’ OR ‘Volatile Organic Compound’OR ‘Volatile Organic Compounds’**OR** ‘**Compounds, Volatile Organic**’ **OR** ‘**Organic Compounds, Volatile**’ **OR**‘**Compound, Volatile Organic**’ **OR** ‘**Organic Compound, Volatile**’):ti,ab,kw | 112,204 |

**Appendix Table 3.** Search strategy - Web of Science

| Search | Query | Results |
| --- | --- | --- |
| #4 | #1 AND #2 AND #3 | 491 |
| #3 | TS=(**"Lung Neoplasms" OR "Lung Cancer" OR "Lung Tumor" OR "Non-Small-Cell Lung" OR "Carcinoma, Non Small Cell Lung" OR "Carcinoma, Non Small Cell Lung" OR "Non-Small-Cell Lung Carcinoma" OR "Non Small Cell Lung Carcinoma" OR "Non-Small Cell Lung Carcinoma" OR "Non-Small Cell Lung Cancer" OR "Carcinoma, Non-Small Cell Lung" OR "Non-small Cell Lung Cancer" OR "Small Cell Lung Cancer"**) | 530,366 |
| #2 | TS=(**"Breath" OR "Exhale" OR "**[**Respiration**](https://www.ncbi.nlm.nih.gov/mesh/68012119)") | 737,053 |
| #1 | TS=(**"Volatile Organic Compounds" OR "Compounds, Volatile Organic" OR "Organic Compounds, Volatile" OR "Volatile Organic Compound" OR "VOC" OR "VOCs" OR "Compound, Volatile Organic" OR "Organic Compound, Volatile"**) | 77,742 |

**Appendix Table 4.** Quality of included studies using the Quality Assessment of Diagnostic Accuracy Studies 2 (QUADAS-2) tool.

| **Study** | **RISK OF BIAS** | | | | **APPLICABILITY CONCERNS** | | |
| --- | --- | --- | --- | --- | --- | --- | --- |
|  | **PATIENT SELECTION** | **INDEX TEST** | **REFERENCE STANDARD** | **FLOW AND TIMING** | **PATIENT SELECTION** | **INDEX TEST** | **REFERENCE STANDARD** |
| Elina et al^1^ | ☹ | ☹ | ☺ | ☺ | ☺ | ☺ | ☺ |
| Michalis et al^2^ | ☹ | ☹ | ☺ | ☹ | ☺ | ☺ | ☺ |
| Tsou et al^3^ | ☹ | ☹ | ☺ | ☺ | ☺ | ☺ | ☺ |
| Long et al^4^ | ☹ | ☹ | ☺ | ☹ | ☺ | ☺ | ☺ |
| Fernanda et al^5^ | ☹ | ☹ | ☺ | ☺ | ☺ | ☺ | ☺ |
| Wang et al^6^ | ☹ | ☹ | ☺ | ☺ | ☺ | ☺ | ☺ |
| Robyn et al^7^ | ☹ | ☹ | ☺ | ☺ | ☹ | ☺ | ☺ |
| Ekaterina et al^8^ | ☹ | ☹ | ☺ | ☺ | ☹ | ☺ | ☺ |
| Azamat Z et al^9^ | ☹ | ☹ | ☺ | ☺ | ☺ | ☺ | ☺ |
| Joanna et al^10^ | ☹ | ☹ | ☺ | ☺ | ☹ | ☺ | ☺ |
| Wang et al^11^ | ☹ | ☺ | ☺ | ☺ | ☺ | ☺ | ☺ |
| Bajtarevic *et al*^12^ | ☹ | ☹ | ☺ | ? | ☺ | ☺ | ☺ |
| Chen *et al*^13^ | ☹ | ☹ | ☺ | ☺ | ☺ | ☺ | ☺ |
| Corradi *et al*^14^ | ☹ | ☹ | ☺ | ☺ | ? | ☺ | ☺ |
| Fuchs *et al*^15^ | ☹ | ☹ | ☺ | ☺ | ☺ | ☺ | ☺ |
| Handa *et al*^16^ | ☹ | ☹ | ☺ | ☺ | ☺ | ☺ | ☺ |
| Phillips *et al*^17^ | ☹ | ☹ | ☺ | ☹ | ☺ | ☺ | ☺ |
| Phillips *et al*^18^ | ☹ | ☹ | ☺ | ☺ | ☺ | ☺ | ☺ |
| Phillips *et al*^19^ | ☹ | ☹ | ☺ | ? | ? | ☺ | ☺ |
| Poli *et al*^20^ | ☹ | ☹ | ☺ | ☺ | ☺ | ☺ | ☺ |
| Sakumura *et al*^21^ | ☹ | ☹ | ☺ | ? | ? | ☺ | ☺ |
| Schallschmidt *et al*^22^ | ☹ | ☹ | ☺ | ☺ | ☺ | ☺ | ☺ |
| Song *et al*^23^ | ☹ | ☹ | ☺ | ☺ | ☺ | ☺ | ☺ |
| Gordon *et al*^24^ | ☹ | ☹ | ☺ | ☺ | ☺ | ☺ | ☺ |
| Fu *et al*^25^ | ☹ | ☹ | ☺ | ☺ | ☹ | ☺ | ☺ |

☺Low Risk ☹High Risk ? Unclear Risk

**Appendix Figure 1.** Risk of bias and applicability concerns using the quality assessment of diagnostic accuracy studies-2 (QUADAS-2) tool.


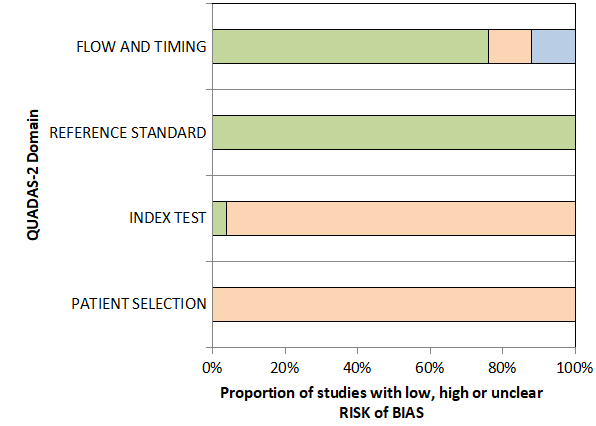

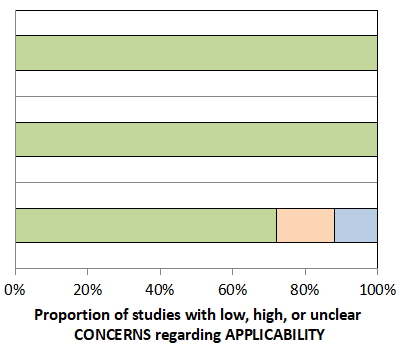

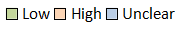


**Appendix Figure 2.** The Positive LR, Negative LR and Diagnostic Odds Ratio of included studies.
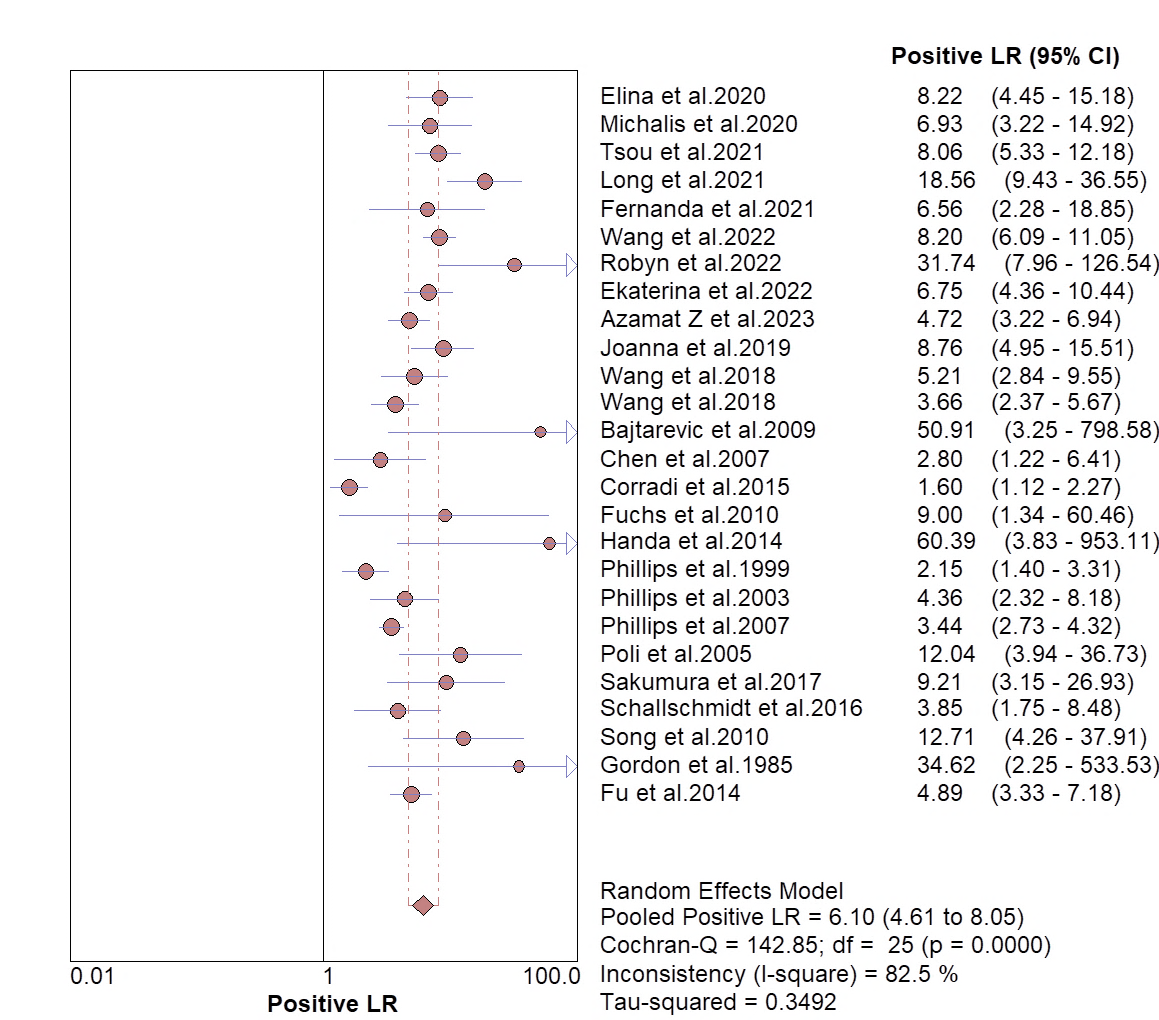

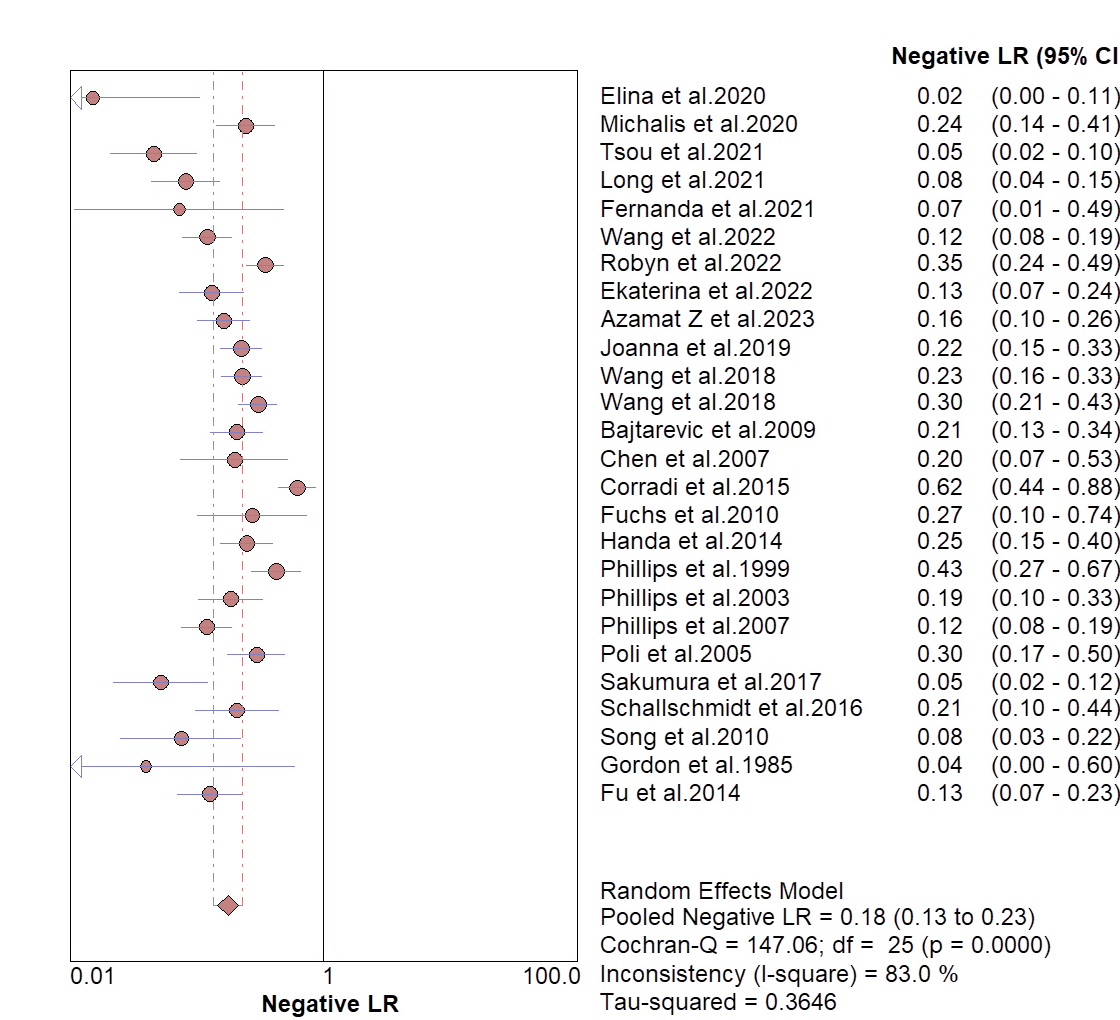


1. B.


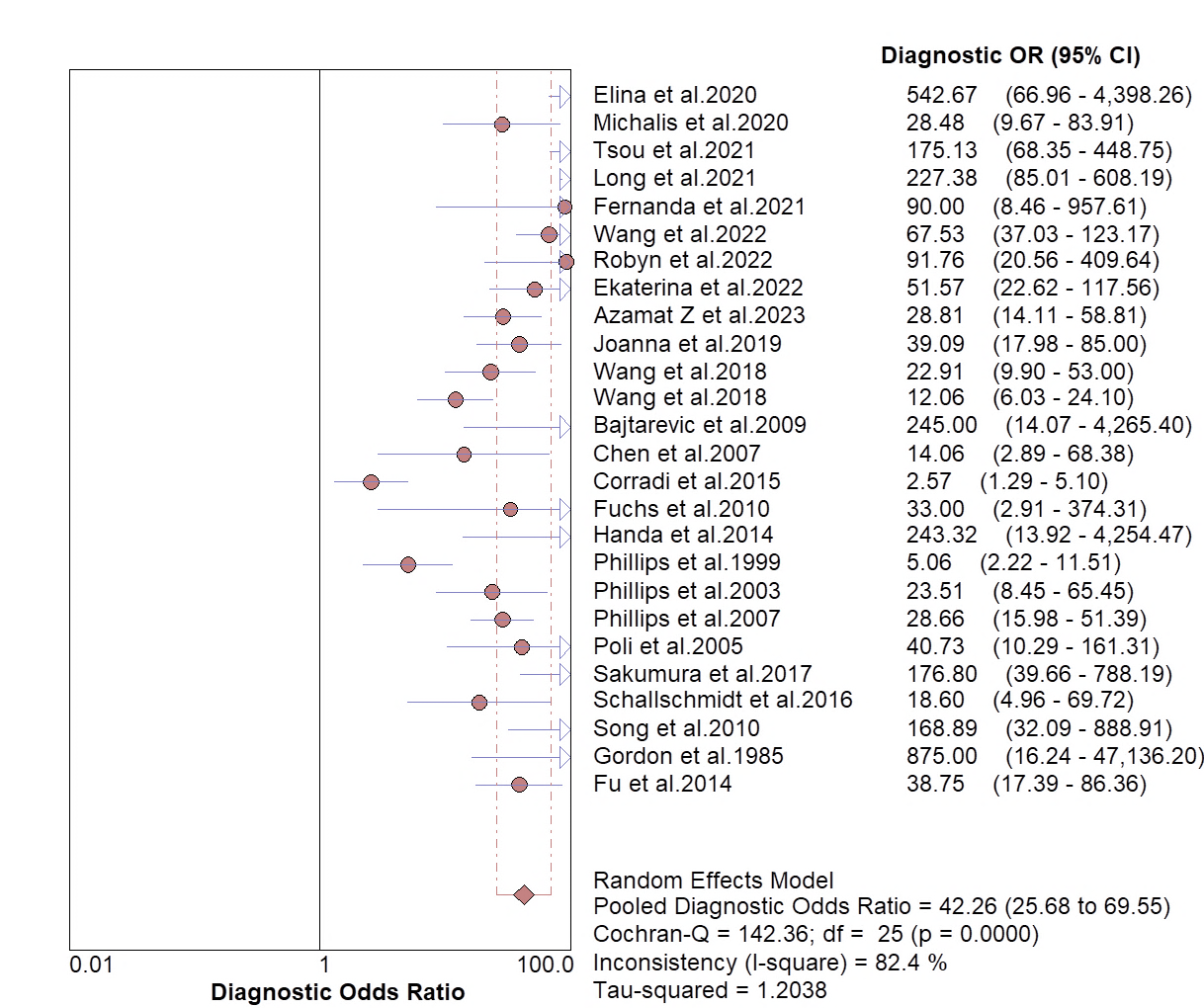


C.

**Appendix Table 5.** Baseline data of the subjects (LC patients and control groups).
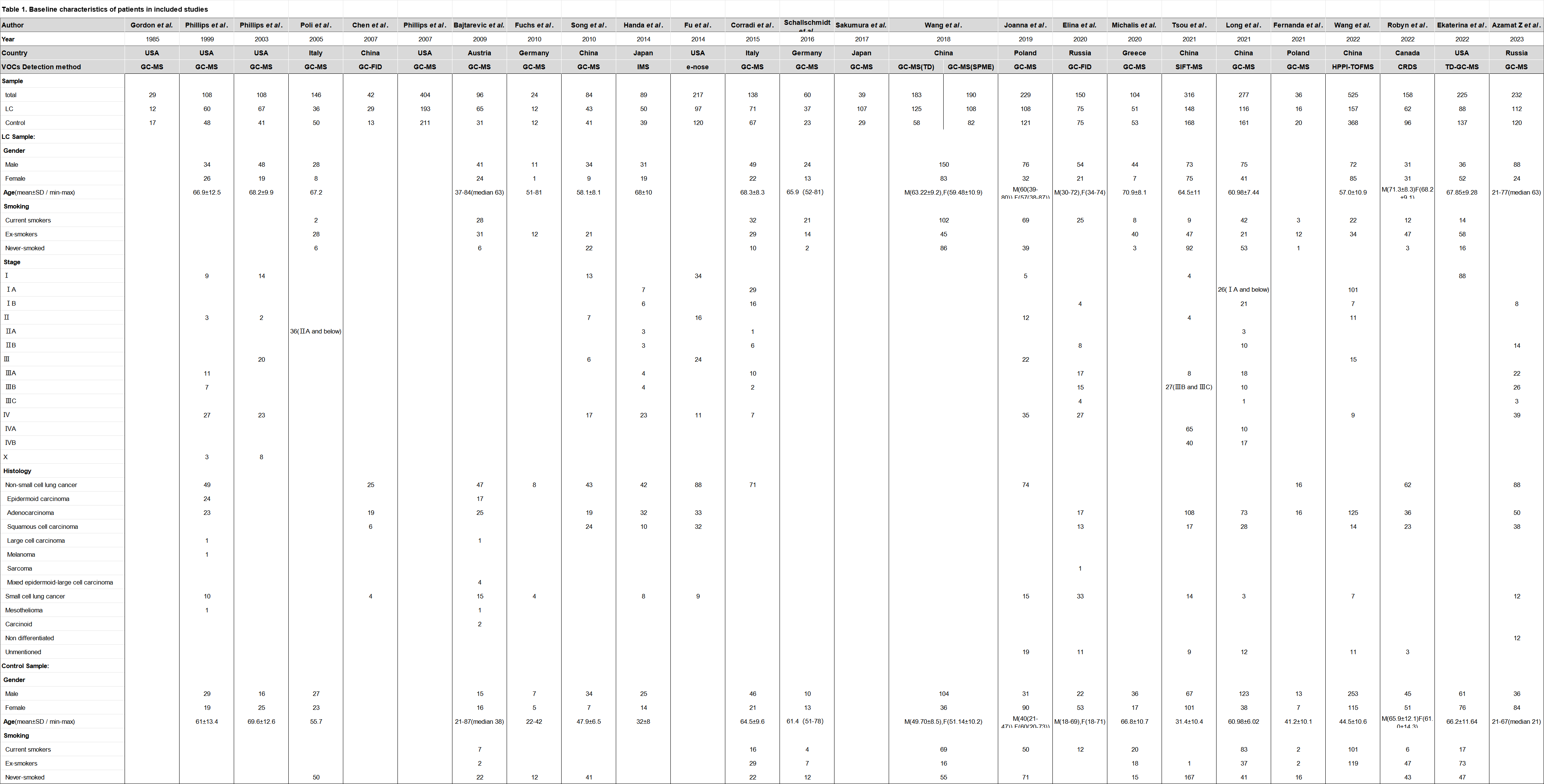


**Appendix Table 6.** VOCs of lung cancer in exhaled breath and their chemical classes.

**References**

1. Gashimova E, Temerdashev A, Porkhanov V, Polyakov I, Perunov D, Azaryan A, Dmitrieva E. Investigation of different approaches for exhaled breath and tumor tissue analyses to identify lung cancer biomarkers. Heliyon. 2020 Jun 17;6(6):e04224.
2. Koureas M, Kirgou P, Amoutzias G, Hadjichristodoulou C, Gourgoulianis K, Tsakalof A. Target Analysis of Volatile Organic Compounds in Exhaled Breath for Lung Cancer Discrimination from Other Pulmonary Diseases and Healthy Persons. Metabolites. 2020 Aug 3;10(8):317.
3. Tsou PH, Lin ZL, Pan YC, Yang HC, Chang CJ, Liang SK, Wen YF, Chang CH, Chang LY, Yu KL, Liu CJ, Keng LT, Lee MR, Ko JC, Huang GH, Li YK. Exploring Volatile Organic Compounds in Breath for High-Accuracy Prediction of Lung Cancer. Cancers (Basel). 2021 Mar 21;13(6):1431.
4. Long Y, Wang C, Wang T, Li W, Dai W, Xie S, Tian Y, Liu M, Liu Y, Peng X, Liu Y, Zhang Y, Wang R, Li Q, Duan Y. High performance exhaled breath biomarkers for diagnosis of lung cancer and potential biomarkers for classification of lung cancer. J Breath Res. 2021 Feb 13;15(1):016017.
5. Monedeiro F, Monedeiro-Milanowski M, Ratiu IA, Brożek B, Ligor T, Buszewski B. Needle Trap Device-GC-MS for Characterization of Lung Diseases Based on Breath VOC Profiles. Molecules. 2021 Mar 22;26(6):1789.
6. Wang P, Huang Q, Meng S, Mu T, Liu Z, He M, Li Q, Zhao S, Wang S, Qiu M. Identification of lung cancer breath biomarkers based on perioperative breathomics testing: A prospective observational study. EClinicalMedicine. 2022 Apr 16;47:101384.
7. Larracy R, Phinyomark A, Scheme E. Infrared cavity ring-down spectroscopy for detecting non-small cell lung cancer in exhaled breath. J Breath Res. 2022 Mar 28;16(2).
8. Smirnova E, Mallow C, Muschelli J, Shao Y, Thiboutot J, Lam A, Rule AM, Crainiceanu C, Yarmus L. Predictive performance of selected breath volatile organic carbon compounds in stage 1 lung cancer. Transl Lung Cancer Res. 2022 Jun;11(6):1009-1018.
9. Temerdashev AZ, Gashimova EM, Porkhanov VA, Polyakov IS, Perunov DV, Dmitrieva EV. Non-Invasive Lung Cancer Diagnostics through Metabolites in Exhaled Breath: Influence of the Disease Variability and Comorbidities. Metabolites. 2023 Jan 30;13(2):203.
10. Rudnicka J, Kowalkowski T, Buszewski B. Searching for selected VOCs in human breath samples as potential markers of lung cancer. Lung Cancer. 2019 Sep;135:123-129.
11. Wang M, Sheng J, Wu Q, Zou Y, Hu Y, Ying K, Wan H, Wang P. Confounding effect of benign pulmonary diseases in selecting volatile organic compounds as markers of lung cancer. J Breath Res. 2018 Sep 14;12(4):046013.
12. Bajtarevic A, Ager C, Pienz M, Klieber M, Schwarz K, Ligor M, Ligor T, Filipiak W, Denz H, Fiegl M, Hilbe W, Weiss W, Lukas P, Jamnig H, Hackl M, Haidenberger A, Buszewski B, Miekisch W, Schubert J, Amann A. Noninvasive detection of lung cancer by analysis of exhaled breath. BMC Cancer. 2009 Sep 29;9:348.
13. Chen X, Xu F, Wang Y, Pan Y, Lu D, Wang P, Ying K, Chen E, Zhang W. A study of the volatile organic compounds exhaled by lung cancer cells in vitro for breath diagnosis. Cancer. 2007 Aug 15;110(4):835-44.
14. Corradi M, Poli D, Banda I, Bonini S, Mozzoni P, Pinelli S, Alinovi R, Andreoli R, Ampollini L, Casalini A, Carbognani P, Goldoni M, Mutti A. Exhaled breath analysis in suspected cases of non-small-cell lung cancer: a cross-sectional study. J Breath Res. 2015 Jan 29;9(2):027101.
15. Fuchs P, Loeseken C, Schubert JK, Miekisch W. Breath gas aldehydes as biomarkers of lung cancer. Int J Cancer. 2010 Jun 1;126(11):2663-70.
16. Handa H, Usuba A, Maddula S, Baumbach JI, Mineshita M, Miyazawa T. Exhaled breath analysis for lung cancer detection using ion mobility spectrometry. PLoS One. 2014 Dec 9;9(12):e114555.
17. Phillips M, Gleeson K, Hughes JM, Greenberg J, Cataneo RN, Baker L, McVay WP. Volatile organic compounds in breath as markers of lung cancer: a cross-sectional study. Lancet. 1999 Jun 5;353(9168):1930-3.
18. Phillips M, Cataneo RN, Cummin AR, Gagliardi AJ, Gleeson K, Greenberg J, Maxfield RA, Rom WN. Detection of lung cancer with volatile markers in the breath. Chest. 2003 Jun;123(6):2115-23.
19. Phillips M, Altorki N, Austin JH, Cameron RB, Cataneo RN, Greenberg J, Kloss R, Maxfield RA, Munawar MI, Pass HI, Rashid A, Rom WN, Schmitt P. Prediction of lung cancer using volatile biomarkers in breath. Cancer Biomark. 2007;3(2):95-109.
20. Poli D, Carbognani P, Corradi M, Goldoni M, Acampa O, Balbi B, Bianchi L, Rusca M, Mutti A. Exhaled volatile organic compounds in patients with non-small cell lung cancer: cross sectional and nested short-term follow-up study. Respir Res. 2005 Jul 14;6(1):71.
21. Sakumura Y, Koyama Y, Tokutake H, Hida T, Sato K, Itoh T, Akamatsu T, Shin W. Diagnosis by Volatile Organic Compounds in Exhaled Breath from Lung Cancer Patients Using Support Vector Machine Algorithm. Sensors (Basel). 2017 Feb 4;17(2):287.
22. Schallschmidt K, Becker R, Jung C, Bremser W, Walles T, Neudecker J, Leschber G, Frese S, Nehls I. Comparison of volatile organic compounds from lung cancer patients and healthy controls-challenges and limitations of an observational study. J Breath Res. 2016 Oct 12;10(4):046007.
23. Song G, Qin T, Liu H, Xu GB, Pan YY, Xiong FX, Gu KS, Sun GP, Chen ZD. Quantitative breath analysis of volatile organic compounds of lung cancer patients. Lung Cancer. 2010 Feb;67(2):227-31. doi: 10.1016/j.lungcan.2009.03.029. Epub 2009 May 5. PMID: 19409642.
24. Gordon SM, Szidon JP, Krotoszynski BK, Gibbons RD, O'Neill HJ. Volatile organic compounds in exhaled air from patients with lung cancer. Clin Chem. 1985 Aug;31(8):1278-82.
25. Fu XA, Li M, Knipp RJ, Nantz MH, Bousamra M. Noninvasive detection of lung cancer using exhaled breath. Cancer Med. 2014 Feb;3(1):174-81.
